# Antenatal *Toxoplasma gondii* IgG/IgM seroprevalence at the University Hospital of Cocody

**DOI:** 10.4102/ajlm.v14i1.2846

**Published:** 2025-12-16

**Authors:** Amah P.V. Goran-Kouacou, Oppong R. Yéboah, Aya U.A. Assi, Yida J. Séri, Séry R. Dassé

**Affiliations:** 1 Laboratory of Immunology and Hematology, University Hospital of Cocody, Abidjan, Côte d’Ivoire; 2 Faculty of Medicine, Félix Houphouët-Boigny University, Abidjan, Côte d’Ivoire

**Keywords:** toxoplasmosis, pregnancy, seroprevalence, antenatal screening, immunodiagnosis, maternal–foetal transmission, Côte d’Ivoire

## Abstract

**Background:**

Toxoplasmosis is a parasitic zoonosis of major importance, particularly during pregnancy because of the risk of maternal–foetal transmission.

**Objective:**

The aim of the study was to estimate the seroprevalence of *Toxoplasma gondii* in pregnant women at the University Hospital of Cocody and to describe IgG/IgM serological profiles.

**Methods:**

We conducted a retrospective cross-sectional study from April 2022 to March 2023 at the immunology laboratory of the University Hospital of Cocody. *Toxoplasma gondii*-specific IgG and IgM antibodies were measured by electrochemiluminescence immunoassay and then interpreted according to serological profiles. Chi-square test was used to assess the association between IgM positivity and pregnancy trimester.

**Results:**

Out of 200 pregnant women, previous infection was observed in 45.0% (IgG^+^/IgM^−^), current infection in 4.0% (IgG^+^/IgM^+^), recent infection in 5.5% (IgG−/IgM^+^), and no infection in 45.5% (IgG^−^/IgM^−^). Of the women, 83% were tested after the first trimester. The proportion of recent infections (IgM^+^) was higher in the first trimester (17.6%) than in the second (8.2%) and third (7.4%) trimesters, with no statistically significant difference (*p* = 0.22).

**Conclusion:**

The seroprevalence of toxoplasmosis remains high, with a non-negligible proportion of women presenting recent or current infection. Late initiation of screening highlights the need for strengthened strategies, including early screening, targeted antenatal education and better access to diagnostic tools to reduce the risk of transmission.

**What this study adds:**

This study provides updated data on the seroprevalence of gestational toxoplasmosis in Côte d’Ivoire. Thus, it reinforces the need for early screening and targeted health awareness campaigns.

## Introduction

Toxoplasmosis is a common zoonotic infection caused by the protozoan *Toxoplasma gondii*. Humans are typically infected by ingesting oocysts that contaminate water or food, or by eating undercooked meat that contains tissue cysts.^1,2^ In immunocompetent individuals, the infection is often asymptomatic, but it can cause serious disease in immunocompromised people and during pregnancy.^3,4^ Primary maternal infection exposes the foetus to vertical (maternal–foetal) transmission, and may lead to severe complications, including neurological and ocular damage, and even death in utero, especially in early infection.^5,6^ Diagnosis relies mainly on serological measurement of *T. gondii*-specific IgG and IgM; IgG-avidity testing and, where indicated, amniotic-fluid polymerase chain reaction (PCR) help to refine the timing of infection.^7,8,9,10,11^ In the absence of pre-existing IgG, pregnant women remain susceptible throughout pregnancy. Although serological screening is recommended as part of antenatal care in several countries, including Côte d’Ivoire, its implementation remains uneven, depending on the context and the resources available.^12,13^ In Africa, toxoplasmosis seroprevalence, typically measured by anti-toxoplasma IgG seropositivity, varies widely among pregnant women, with rates of 44.4% in Cotonou,^14^ 36.1% in rural Benin,^13^ and 51% in Morocco.^15^ In Côte d’Ivoire, data are scarce, with the most recent study in Abidjan reporting a seroprevalence rate of 48%,^16^ underlining the high level of endemicity. However, these data appear outdated and require re-evaluation, particularly owing to the continued presence of environmental and dietary risk factors. This study was therefore designed to estimate the seroprevalence of toxoplasmosis and to describe IgG/IgM serological profiles in pregnant women attending the University Hospital of Cocody.

## Methods

### Ethical considerations

The study was carried out with respect for the confidentiality of the data. The information used came exclusively from the laboratory registers and did not include any nominative elements. The study was performed in accordance with the ethical principles of biomedical research and received the approval of the institutional ethics committee (reference number: 042/MSHP-CMU/CHU-C/DMS/RK/25) of the University Hospital of Cocody. The anonymity and confidentiality of the data were guaranteed.

### Study design

A retrospective cross-sectional study of serological data from pregnant women was conducted between April 2022 and March 2023.

### Study location

The study took place in the immunology laboratory at the University Hospital of Cocody. This laboratory is a reference centre for prenatal immunological examinations in the region.

### Study population and selection criteria

The study involved pregnant women who had undergone toxoplasma serology as part of their antenatal check-up or as a follow-up to a previous serology. Patients for whom the following data were available were included: serological results (IgG and IgM), maternal age and gestational age. Files with missing data were excluded.

### Data collection

Data were collected from laboratory records using a standardised form. The variables collected included the patients’ age in years, gestational age in weeks of gestation, the reason for the request (routine screening or follow-up), and the results of specific IgG and IgM assays for *T. gondii*. In accordance with obstetric recommendations, the trimesters were divided as follows: first trimester (≤ 14 weeks of gestation), second trimester (15–27 weeks of gestation) and third trimester (≥ 28 weeks of gestation). Data regarding any treatment and obstetric or neonatal outcomes are not recorded in laboratory registers and were therefore unavailable for analysis.

### Laboratory tests

IgG and IgM serologies were analysed by sandwich electrochemiluminescence immunoassay on a Cobas 6000 e601 (Roche Diagnostics GmbH, Mannheim, Germany), using Elecsys^®^ Toxo IgG and IgM reagents. The interpretation thresholds, according to the manufacturer’s recommendations, were as follows: specific IgG was considered negative (< 1 IU/mL), equivocal (1 IU/mL – < 3 IU/mL), or positive (≥ 3 IU/mL); specific IgM was considered negative (cut-off index: < 0.8), equivocal (cutt-off index: 0.8 – < 1.0), or positive (cut-off index: ≥ 1.0).

### Serological interpretation

Based on IgG and IgM status, four serological profiles were defined: previous infection (IgG^+^/IgM^–^), current infection (IgG^+^/IgM^+^), recent infection (IgG^–^/IgM^+^), and no infection (IgG^–^/IgM^–^).^17^ Equivocal results were interpreted according to the manufacturer’s instructions and were not used for prevalence calculations.

### Data analysis

Data were entered and processed using Microsoft Excel 2016. Statistical analyses were performed using SPSS version 26.0. Descriptive analysis summarised the sociodemographic, obstetric and serological characteristics of the patients, with results presented in absolute numbers and percentages. Comparative analysis was used to explore the association between IgM positivity and the trimester of pregnancy, using Pearson’s chi-squared (χ^2^) test; *p* < 0.05 was considered statistically significant.

## Results

### General characteristics of the population

Data were analysed from 200 pregnant women aged 18–44 years, with a predominance in the 25–34 age group (44.0%), followed by those younger than 25 years (31.5%) (Table 1). The majority were in the second trimester (42.5%), compared with 40.5% in the third and 17.0% in the first (Table 1).

**TABLE 1:** Distribution of pregnant women according to demographic, clinical and serological data, Immunology Laboratory, University Hospital of Cocody, Abidjan, Côte d’Ivoire, April 2022 – March 2023.

Variables	*n*	%
**Age (years)**		
< 25	63	31.5
25–34	88	44.0
≥ 35	49	24.5
**Gestational age**		
First trimester (≤ 14 WG)	34	17.0
Second trimester (15–27 WG)	85	42.5
Third trimester (≥ 28 WG)	81	40.5
**Reason for serological request**		
Antenatal screening	169	84.5
Follow-up	31	15.5

WG, weeks of gestation.

### Serological distribution

Based on *T. gondii* IgG/IgM results, four serological profiles were identified (Table 2: previous infection, current infection, recent infection, and no infection). Overall IgG seroprevalence was 49.0% (45.0% IgG^+^/IgM^−^; 4.0% IgG^+^/IgM^+^). Nineteen women (9.5%) had IgM-positive profiles, indicating recent or current infection. IgM positivity was most frequent in the first trimester (17.6%), followed by the second (8.2%) and third trimesters (7.4%) (Table 3), with no statistically significant difference (χ^2^ = 2.99; *df* = 2; *p* = 0.22).

**TABLE 2:** Distribution of immunological profiles based on IgG and IgM results for *Toxoplasma gondii*, Immunology Laboratory, University Hospital of Cocody, Abidjan, Côte d’Ivoire, April 2022 – March 2023.

IgG/IgM profile	Immunological interpretation	*n*	%
IgG^+^/IgM^−^	Previous infection	90	45.0
IgG^+^/IgM^+^	Current infection	8	4.0
IgG^−^/IgM^+^	Recent infection	11	5.5
IgG^−^/IgM^−^	No infection	91	45.5

IgG, immunoglobulin G; IgM, immunoglobulin M.

**TABLE 3:** Distribution of IgM-positive profiles according to the age of pregnancy, Immunology Laboratory, University Hospital of Cocody, Abidjan, Côte d’Ivoire, April 2022 – March 2023.

Trimester of pregnancy	IgM^+^	*n*	%
First trimester	6	34	17.6
Second trimester	7	85	8.2
Third trimester	6	81	7.4

Note: χ^2^ = 2.99; *df* = 2; *p* = 0.22.

*df*, degrees of freedom; IgM, immunoglobulin M.

### Reason for requesting serology

Toxoplasma serology was carried out as part of routine prenatal screening in 169 women (84.5%) and as part of the follow-up of patients at risk in 31 women (15.5%) (Table 1). Of the 169 screened, only 29 (17.2%) were tested in the first trimester, compared with 74 (43.8%) in the second and 66 (39.0%) in the third (Table 4).

**TABLE 4:** Distribution of requests for toxoplasma serology according to the age of pregnancy and the reason for carrying out the test, Immunology Laboratory, University Hospital of Cocody, Abidjan, Côte d’Ivoire, April 2022 – March 2023.

Trimester of pregnancy	Screening		Follow-up		Total	
	*n*	%	*n*	%	*n*	%
First trimester	29	17.1	5	16.1	34	17.0
Second trimester	74	43.8	11	35.4	85	42.5
Third trimester	66	39.1	15	48.3	81	40.5

**Total**	**169**	**100**	**31**	**100**	**200**	**100**

## Discussion

The results of this study show an overall IgG seroprevalence of 49.0% among pregnant women. This rate is comparable to those reported in Côte d’Ivoire in Abidjan (48%),^16^ and in Daloa (54.16%),^18^ as well as in Benin in Cotonou (44.4%),^14^ and in rural areas (36.1%),^13^ indicating sustained *T. gondii* endemicity in West Africa. Similar rates have also been observed in other tropical contexts, such as Mayotte^19^ and Morocco.^15^ At the individual level, previous infection (IgG^+^/IgM^−^) is generally considered reassuring for the current pregnancy. However, the significant proportion of women with positive IgM (9.5%), including 5.5% with IgG^−^/IgM^+^ and 4.0% with IgG^+^/IgM^+^, is of concern. These serological profiles reflect recent or current infection, with a risk of foetal transmission, particularly during the first two trimesters.^6,20^ In the absence of an IgG-avidity test, it remains difficult to distinguish acute infection, reinfection or persistence of IgM.^9,10^ However, several authors recommend that, in cases of IgM positivity, treatment with spiramycin be initiated early, accompanied by close ultrasound monitoring, even in the absence of confirmation by IgG-avidity testing.^12^

Furthermore, a substantial proportion of women with no infection (IgG^−^/IgM^−^; 45.5%) constitutes a susceptible group lacking protective IgG. Although exposure pathways were not assessed in this study, targeted antenatal counselling on food and environmental hygiene, including thorough cooking of meat, remains essential to prevent primary infection during pregnancy. In a highly endemic context such as Côte d’Ivoire,^3,21^ this risk must be considered actively. Evidence indicates that the risk of vertical transmission rises with advancing gestation, from 10% – 15% in the first trimester to more than 60% in the third, although the most severe clinical forms of congenital toxoplasmosis are linked to early infections.^22^

Cross-analysis between pregnancy trimesters and serological profiles showed that IgM-positive cases occurred in all trimesters, with a higher proportion in the first (17.6%) than in the second (8.2%) and third (7.4%). Although this difference did not reach statistical significance (χ^2^ = 2.99; *df* = 2; *p* = 0.22), the pattern is consistent with a higher risk of primary infection in early pregnancy, a critical period for the foetus. These results, although observed in the absence of ultrasound or neonatal confirmation, underscore the need for increased vigilance from the first trimester, including, where possible, amniotic-fluid PCR when a recent infection is suspected.^11,12,23^ Our work is a seroprevalence study based on laboratory data; the assessment of clinical management and obstetric or neonatal outcomes was not part of the present analysis.

Furthermore, our study revealed late initiation of toxoplasmosis screening among pregnant women. Only 17.2% were screened in the first trimester, compared with 43.8% and 39.0% in the second and third trimesters, respectively. This delay limits opportunities for early diagnosis and, where indicated, timely preventive measures. In Côte d’Ivoire, first trimester screening is recommended, but uptake is constrained by out-of-pocket payment, as the test is not covered routinely. This financial barrier delays early screening and supports consideration of measures to improve affordability, for example, through targeted fee exemptions or inclusion of toxoplasmosis serology in the essential antenatal-care package.

The main limitation of this study lies in its retrospective design without longitudinal follow-up. Seroconversions and pregnancy outcomes (confirmation of foetal infection or neonatal serology) could not be documented.^24,25^ In addition, clinical management data (e.g. treatment initiation) and obstetric or neonatal outcomes were not recorded in the laboratory registers and were therefore unavailable for analysis, which, together with the absence of IgG-avidity testing and amniotic-fluid PCR, limits precise dating of infection phases and the clinical interpretation of the findings.^11,12^ Although IgG titres were recorded in the laboratory registers, they were not analysed here because their interpretation remains difficult without avidity testing; however, prior reports suggest that very high IgG titres may indicate long-standing immunity.^10,16^

Despite these limitations, our results support strengthening prenatal monitoring practices: the implementation of systematic screening from the first trimester, serological testing in cases of seronegativity or clinical doubt, and better interpretation of serological profiles. Countries such as France have demonstrated that early treatment with spiramycin reduces the foetal transmission rate significantly from more than 30% to less than 5%.^23^ In Côte d’Ivoire, although national recommendations exist, their application remains inconsistent. It is therefore essential to train medical personnel in serological interpretation, to make IgG-avidity and PCR testing available, to raise awareness among pregnant women about preventive measures, and to ensure access to specific treatments.

### Conclusion

Gestational toxoplasmosis remains a concern in our setting. A notable proportion of pregnant women had serological evidence of recent or current infection. Screening frequently begins after the first trimester, limiting opportunities for early diagnosis and timely preventive management. These findings support initiating screening in early pregnancy, strengthening targeted antenatal counselling on food and environmental hygiene, and improving access to confirmatory diagnostics (e.g. IgG-avidity testing and, where indicated, amniotic-fluid PCR).
